# Intraindividual reproducibility of myocardial radiomic features between energy-integrating detector and photon-counting detector CT angiography

**DOI:** 10.1186/s41747-024-00493-7

**Published:** 2024-08-28

**Authors:** Giuseppe Tremamunno, Akos Varga-Szemes, U. Joseph Schoepf, Andrea Laghi, Emese Zsarnoczay, Nicola Fink, Gilberto J. Aquino, Jim O’Doherty, Tilman Emrich, Milan Vecsey-Nagy

**Affiliations:** 1 Department of Radiology and Radiological Science, Medical University of South Carolina, Charleston, SC USA; 2 Department of Medical Surgical Sciences and Translational Medicine, Sapienza University of Rome, Rome, Italy; 3 Medical Imaging Centre, Semmelweis University, Budapest, Hungary; 4 Department of Radiology, University Hospital, LMU Munich, Munich, Germany; 5 Siemens Medical Solutions, Malvern, PA USA; 6 Department of Diagnostic and Interventional Radiology, University Medical Center of the Johannes Gutenberg-University Mainz, Mainz, Germany; 7 Heart and Vascular Center, Semmelweis University, Budapest, Hungary

**Keywords:** Computed tomography angiography, Myocardium (radiomics), Reproducibility of results, Tomography (x-ray computed)

## Abstract

**Background:**

Radiomics is not yet used in clinical practice due to concerns regarding its susceptibility to technical factors. We aimed to assess the stability and interscan and interreader reproducibility of myocardial radiomic features between energy-integrating detector computed tomography (EID-CT) and photon-counting detector CT (PCD-CT) in patients undergoing coronary CT angiography (CCTA) on both systems.

**Methods:**

Consecutive patients undergoing clinically indicated CCTA on an EID-CT were prospectively enrolled for a PCD-CT CCTA within 30 days. Virtual monoenergetic images (VMI) at various keV levels and polychromatic images (T3D) were generated for PCD-CT, with image reconstruction parameters standardized between scans. Two readers performed myocardial segmentation and 110 radiomic features were compared intraindividually between EID-CT and PDC-CT series. The agreement of parameters was assessed using the intraclass correlation coefficient and paired *t*-test for the stability of the parameters.

**Results:**

Eighteen patients (15 males) aged 67.6 ± 9.7 years (mean ± standard deviation) were included. Besides polychromatic PCD-CT reconstructions, 60- and 70-keV VMIs showed the highest feature stability compared to EID-CT (96%, 90%, and 92%, respectively). The interscan reproducibility of features was moderate even in the most favorable comparisons (median ICC 0.50 [interquartile range 0.20–0.60] for T3D; 0.56 [0.33–0.74] for 60 keV; 0.50 [0.36–0.62] for 70 keV). Interreader reproducibility was excellent for the PCD-CT series and good for EID-CT segmentations.

**Conclusion:**

Most myocardial radiomic features remain stable between EID-CT and PCD-CT. While features demonstrated moderate reproducibility between scanners, technological advances associated with PCD-CT may lead to greater reproducibility, potentially expediting future standardization efforts.

**Relevance statement:**

While the use of PCD-CT may facilitate reduced interreader variability in radiomics analysis, the observed interscanner variations in comparison to EID-CT should be taken into account in future research, with efforts being made to minimize their impact in future radiomics studies.

**Key Points:**

- Most myocardial radiomic features resulted in being stable between EID-CT and PCD-CT on certain VMIs.
- The reproducibility of parameters between detector technologies was limited.
- PCD-CT improved interreader reproducibility of myocardial radiomic features.

**Graphical Abstract:**

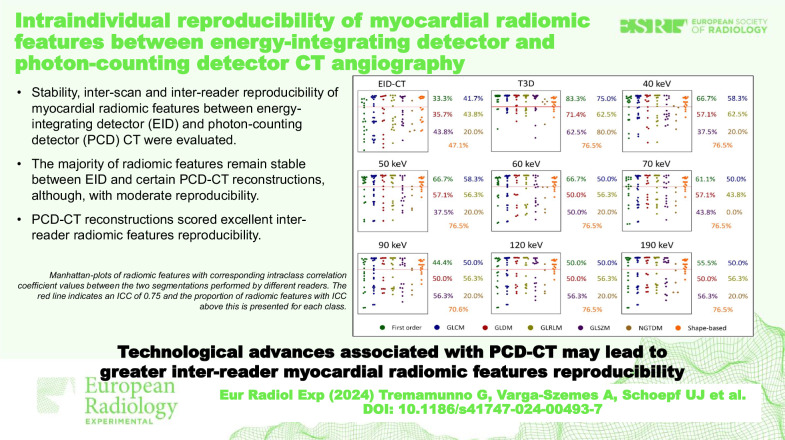

## Background

Radiomics has become a promising area of research in precision medicine, providing opportunities to facilitate disease phenotyping based on radiological images [1]. By extracting and mathematically processing multiple features from a single volume of interest, it is possible to create predictive models that may potentially be beneficial in diagnostics, predicting outcomes, or selecting treatment individually [2–5]. Several studies have shown that exploiting the potential of radiomic analysis can improve the diagnostic accuracy of coronary computed tomography angiography (CCTA) and may overcome the limitations of qualitative evaluation, which is influenced by the experience of the individual reader [6, 7]. Radiomic models have demonstrated efficacy in detecting advanced atherosclerotic lesions, and plaque vulnerability markers—such as the napkin-ring sign—and in confirming the pericoronary fat radiomic profile as an independent predictor of major adverse cardiovascular events [8–10]. Recently, the ability of texture-based radiomic models has also been proposed to differentiate between healthy and infarcted myocardium, and identify patients at high risk of left ventricular hypertrophy and those with recurrent ventricular tachycardia [11–13]. Despite its advantages, radiomics are not yet widely used in clinical practice due to concerns regarding its susceptibility to a number of technical factors and the effect of segmentation performed by different readers [14–18].

Recently, studies have demonstrated multiple benefits of photon-counting detector computed tomography (PCD-CT) in comparison to conventional energy-integrating detector computed tomography (EID-CT), such as enhanced contrast-to-noise ratio and increased spatial resolution, which are crucial image quality factors that ensure the accuracy of texture analysis [16, 19, 20]. However, it remains unclear whether and to what extent this new detector technology affects the stability of texture features, and a systematic comparison with conventional EID-CT may bring standardization and eventual validation of the most robust features.

Therefore, this study aimed to evaluate the impact of novel PCD-CT technology on the stability, interscanner reproducibility, and interreader reproducibility of myocardial radiomic features through a systematic intraindividual comparison of a cohort of patients undergoing CCTA on both EID-CT and PCD-CT. Given the study design, it was hypothesized that any discrepancies observed in radiomic parameters could be attributed primarily to differences in detector technology.

## Methods

### Patient population

The protocol for this single-center, prospective study was approved by the local Institutional Review Board (see “Declarations”). It was conducted in accordance with the tenets of the Declaration of Helsinki and all subjects provided written informed consent. Consecutive patients referred for standard-of-care imaging on an EID-CT system were asked to be enrolled for a research PCD-CT scan within 30 days, between August 2021 and March 2022. Patients over 18 years of age undergoing clinically indicated CCTA met the inclusion criteria. Exclusion criteria were as follows: contraindication to iodinated contrast media, decreased renal function (glomerular filtration rate < 45 mL/min/m^2^), pregnancy or lactation, inability to consent, and inconsistent acquisition modes between scans.

### CCTA acquisition parameters

First, a clinical CCTA was carried out using a third-generation dual-source EID-CT (SOMATOM Force, Siemens Healthineers, Forchheim, Germany). Research CCTA was subsequently performed using a first-generation clinical dual-source PCD-CT system (NAEOTOM Alpha, Siemens). EID-CT scans were carried out using a high-pitch or sequential cardiac protocol, while all PCD-CT datasets were acquired in sequential mode with end-diastolic ECG triggering. As per the standard clinical protocol, the tube voltage for EID-CT was automatically determined by the scanner at 90 kVp, 110 kVp, or 130 kVp, depending on the patient’s body habitus. For PCD-CT, tube voltage was manually set at 120 kVp. Automatic tube current modulation (CareDose 4D, Siemens) was used for EID-CT, while the tube current for PCD-CT acquisition was adjusted to closely match the expected radiation dose (volume CT dose index) between the two scans. Scan parameters are summarized in Table 1.

**Table 1:** CCTA acquisition and reconstruction parameters

Parameters	EID-CT	PCD-CT
Tube potential (kVp)		
90, *n*	1	–
110, *n*	10	–
120, *n*	–	18
130, *n*	7	–
Tube current (mAs)	262.5 (187.5–298.5)	Image quality level = 64
Rotation time (s)	0.25	0.25
Temporal resolution (ms)	66	66
Reconstruction energy threshold	Polychromatic	Polychromatic (T3D) and 40 keV, 50 keV, 60 keV, 70 keV, 90 keV, 120 keV, 190 keV
Iterative reconstruction (level)	ADMIRE (2)	QIR (2)
Reconstruction kernel	Bv40	Bv40
Slice thickness (mm)	0.6	0.6
Slice increment (mm)	0.5	0.5
Matrix size	512 × 512	512 × 512

*ADMIRE* Advanced modeled iterative reconstruction, *CT* Computed tomography, *EID-CT* Energy-integrating detector CT, *PCD-CT* Photon-counting detector CT, *QIR* Quantum iterative reconstruction, *VMI* Virtual monoenergetic reconstruction

The contrast administration protocol used was identical for both scanners. Based on our institution’s standard clinical injection protocol, patients were administered a triphasic injection comprising an initial bolus of 40–60 mL of iodinated contrast agent (Omnipaque 350, GE Healthcare, Boston, MA, or Ultravist 370, Bayer, Berlin, Germany), followed by a 50% dilute mixture of contrast agent and saline (20 mL), and finally a saline chaser (25 mL). The volume of contrast material was kept constant between EID-CT and PCD-CT scans, at a constant injection rate (3.5–5.0 mL/s). Unless clinically contraindicated, patients with heart rates > 70 beats per minute received intravenous administration of 5 mg metoprolol and approximately 0.4 mg nitroglycerin 5 min before the scan.

### Image reconstruction

Images from EID-CT and PCD-CT were reconstructed as equivalent as possible at a slice thickness of 0.6 mm, using a Bv40 vascular kernel, a matrix size of 512 × 512, and a consistent field of view between scanners, as reported in Table 1. Virtual monoenergetic images (VMI) were reconstructed on PCD-CT at seven different levels (40 keV, 50 keV, 60 keV, 70 keV, 90 keV, 120 keV, and 190 keV). Additionally, “T3D” (Siemens Healthineers) were reconstructed for PCD-CT, which are considered most comparable to a conventional polychromatic reconstruction on EID-CT systems, as they accumulate all x-ray photons with energies above the lowest threshold energy of 20 keV and therefore integrate polychromatic information [21]. A similar level of iterative reconstruction was used for EID-CT and PCD-CT, with a strength level of 2 for advanced modeled iterative reconstruction and quantum iterative reconstruction, respectively.

### Segmentation of the left ventricle

The image datasets were exported and segmented using investigational software (MM Radiomics v1.4.0, Siemens). Although segmentation was performed manually, a threshold-assisted segmentation tool was employed, with the exclusion of voxels outside the specified threshold interval of the myocardium (from −30 HU to 250 HU), in order to exclude adjacent tissues (epicardial fat, blood pool) from the analysis. Subsequently, the ‘Fill Cavities’ function was applied to the segmented volumes, resulting in a further augmentation of the volume by all voxels within the object that are not connected to the external surfaces, based on a three-dimensional six-neighborhood relationship. The segmentation area included the trabecular and papillary muscles as well. The segmentations were performed by two readers with 4 years and 6 years of experience in cardiovascular CT and the segmentation of the more experienced reader was used for final analysis. If necessary, the delineation of boundaries was corrected by software-specific refinement tools. For patients scanned on the PCD-CT system, segmentations were performed using the 70-keV series, and the resulting volume of interest was transferred to the other six VMI reconstructions and the T3D dataset.

### Feature extraction

A total of 110 radiomic features were generated for each dataset using the same investigational software (MM Radiomics, Siemens). The extracted feature classes consisted of original first-order (*n* = 18), textural [gray level co-occurrence matrix (GLCM), *n* = 24; gray level dependence matrix (GLDM), *n* = 14; gray level run length matrix (GLRLM), *n* = 16; gray level size zone matrix (GLSZM), *n* = 16; and neighboring gray-tone difference matrix (NGTDM), *n* = 5], as well as two- and three-dimensional-based shape features (*n* = 17). In total, 17,820 original myocardial radiomic features were extracted from the datasets. Detailed mathematical descriptions of feature families and their corresponding feature members are available elsewhere [22].

### Statistical analysis

**Fig. 1:**
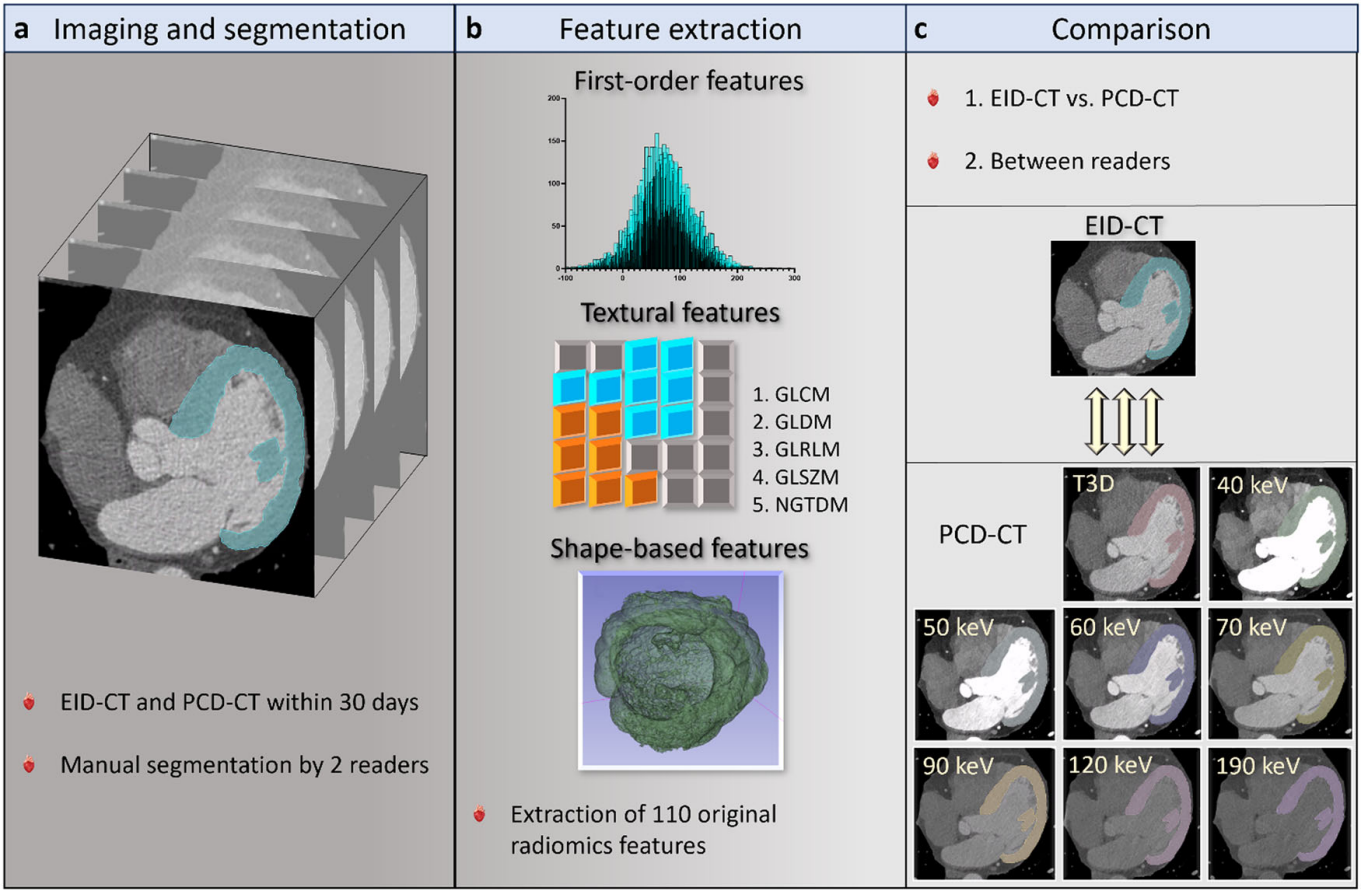
The overall acquisition, processing, and analysis framework of the datasets. Both EID-CT and PCD-CT datasets of enrolled patients were segmented (**a**) by two readers. A total of 18 first-order, 75 textural, and 17 shape-based radiomic features were extracted (**b**) from the segmented volume of interests. These values were then compared (**c**) between EID-CT and PCD-CT reconstructions, among different VMIs, and between different segmentations to assess inter-reader agreement. EID-CT, Energy-integrating detector CT; GLCM, Gray level co-occurrence matrix; GLDM, Gray level dependency matrix; GLRLM, Gray level run length matrix; GLSZM, Gray level size zone matrix; NGTDM, Neighboring gray-tone difference matrix; PCD-CT, Photon-counting detector CT; VMI, Virtual monoenergetic image

Normally distributed variables are reported as mean ± standard deviation, while those with non-normal distribution are expressed as median and 25th–75th percentiles. Categorical variables are reported as absolute frequencies and proportions. The Kolmogorov–Smirnov test was applied to evaluate the normality of continuous parameters. Normally distributed descriptive statistics were compared using paired *t*-tests, while non-normally distributed variables were compared using the Wilcoxon test. Pairwise comparisons between myocardial radiomic features of EID-CT and PCD-CT were performed with paired samples *t*-test; *p*-values < 0.05 were regarded as statistically significant and interpreted as indicative of unstable features between EID-CT- and PCD-CT-based reconstructions (VMI and T3D). The percentage of features that exhibit non-significant pairwise differences was evaluated to assess the stability across each PCD-CT series and between PCD-CT and EID-CT datasets. Shape class features were excluded in the comparison between all VMIs and T3D images acquired on PCD-CT, since the same volume of interest was transferred across all reconstructions. A two-way mixed intraclass correlation coefficient (ICC) was used to assess the overall degree of agreement between datasets and the segmentations performed by the two readers. ICC was interpreted as follows: poor (ICC ≤ 0.39), moderate (ICC = 0.40–0.59), good (ICC = 0.60–0.74), and excellent (ICC ≥ 0.75) [23]. Manhattan plots were generated to graphically display both results. Statistical analysis was performed using dedicated software (SPSS Statistics, version 27.0, IBM Corporation, Armonk, NY, USA; MedCalc, version 20.2, San Diego, CA, USA). The stepwise summary of the analysis is provided in Fig. 1.

## Results

### Patient population

A total of 24 patients were enrolled consecutively and scanned on both EID-CT and PCD-CT. Overall, six scans were excluded from the final analysis due to inconsistent acquisition modes between the scanners (EID-CT, high-pitch helical; PCD-CT, sequential). The final cohort consisted of 18 patients aged 67.6 ± 9.7 years (mean ± standard deviation), and 15 males (83.3%). The median time interval between the two scans was 7.0 days (interquartile range [IQR] 3.0–15.0). While heart rate did not differ significantly between EID-CT and PCD-CT, PCD-CT was performed with significantly lower volume CT dose index and dose length product values. Further details are summarized in Table 2.

**Table 2:** Patient characteristics

	EID-CT	PCD-CT	*p*-value
Number	18		
Male	15		
Age (years)	67.6 ± 9.7		
BMI (kg/m^2^)	31.5 ± 7.7		
Heart rate (L/min)	67.1 ± 15.5	64.8 ± 13.5	0.638
CTDI_vol_ (mGy)	39.1 (25.8–58.5)	29.9 (24.2–39.6)	0.010
DLP (mGy × cm)	469.5 (344.8–913.8)	442.0 (342.8–584.5)	0.040

Data are given as mean ± standard deviation or median (interquartile range) according to their normal or non-normal distribution

*BMI* Body mass index, *CTDI*_*vol*_ Volume CT dose index, *DLP* Dose length product, *EID-CT* Energy-integrating detector CT, *PCD-CT* Photon-counting detector CT

### Stability of radiomic features

The stability of features across datasets was evaluated by calculating the percentage of features that exhibit non-significant pairwise differences. Across different VMIs on PCD-CT, a number of comparisons produced a high percentage of stable features (40 keV *versus* 50 keV, 96%; 50 keV *versus* 60 keV, 96%; 60 keV *versus* 70 keV, 96%; 70 keV *versus* 90 keV, 95%; 70 keV *versus* 120 keV, 92%; 70 keV *versus* 190 keV, 92%; 90 keV *versus* 120 keV, 98%; 90 keV *versus* 190 keV, 95%; 120 keV *versus* 190 keV, 98%). The polychromatic (T3D) PCD-CT reconstruction produced the highest number of stable features in comparison to 50 keV (T3D *versus* 50 keV, 92%) and 60 keV (T3D *versus* 60 keV, 100%).

**Fig. 2:**
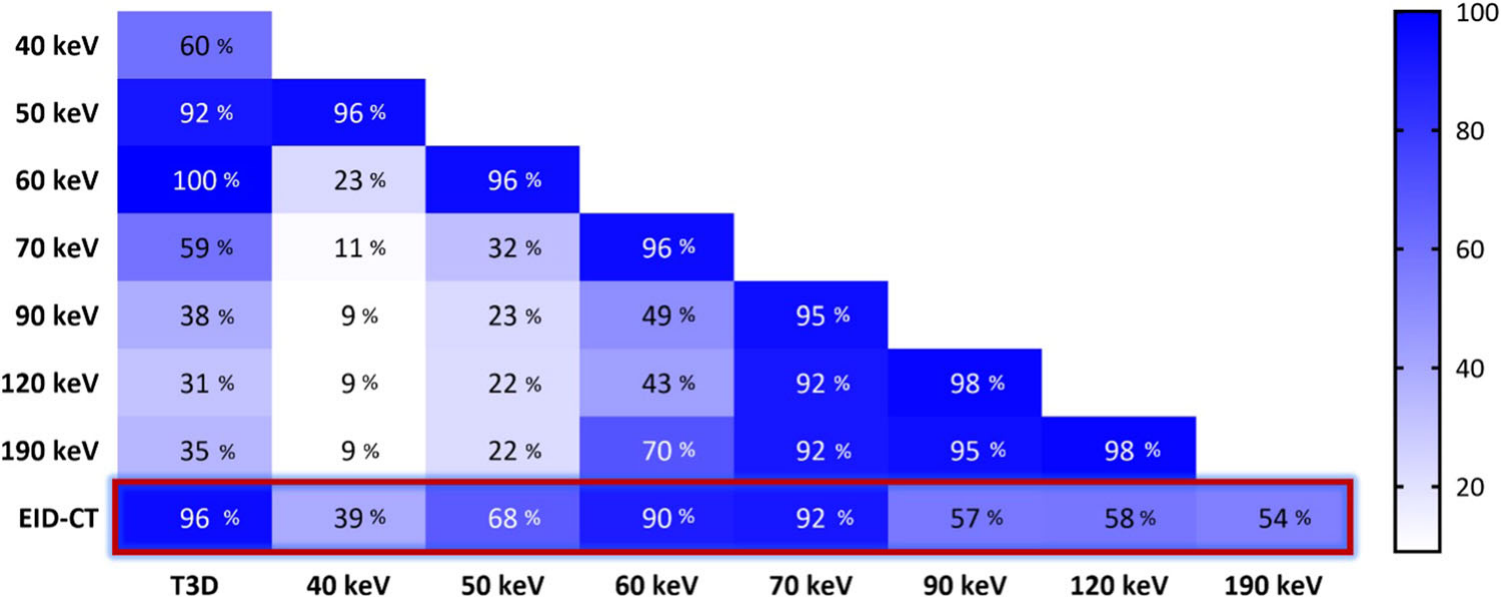
Heatmap of the proportion of stable features (without a significant difference) between pairwise comparisons. A high number of features demonstrated stability for EID-CT compared to T3D, 60 keV and 70 keV PCD-CT reconstructions. Several features showed non-significant differences between different VMI levels as well, with an increasingly favorable trend at higher VMI levels. EID-CT, Energy-integrating detector CT; PCD-CT, Photon-counting detector CT

In terms of feature reproducibility between scanners, EID-CT showed the highest similarity to polychromatic PCD-CT reconstruction (EID-CT *versus* T3D, 96%), with only four features differing significantly between them (firstorder_Median, gldm_GrayLevelNonUniformity, grlm_GrayLevelNonUniformity, ngtdm_Coarseness). Across different VMI levels, 60 keV (EID-CT *versus* 60 keV, 90%) and 70 keV (EID-CT *versus* 70 keV, 92%) demonstrated a high proportion of stable features, with 70 keV showing significant differences in only nine features (firstorder_Median, Mean and firstorder_10Percentile, gldm_DependenceNonUniformity, glrlm_RunLengthNonUniformity and GrayLevelNonUniformity, glszm_GrayLevelNonUniformity, and SizeZoneNonUniformity, ngtdm_Coarseness). Among all 110 original radiomic features, two were statistically different between EID-CT and all PCD-CT reconstructions: firstorder_Median and ngtdm_Coarseness. Figure 2 details the proportion of stable features in all pairwise comparisons, while an overview of all features with significant differences is presented in Supplementary Table S1.

### Reproducibility of radiomic features

**Fig. 3:**
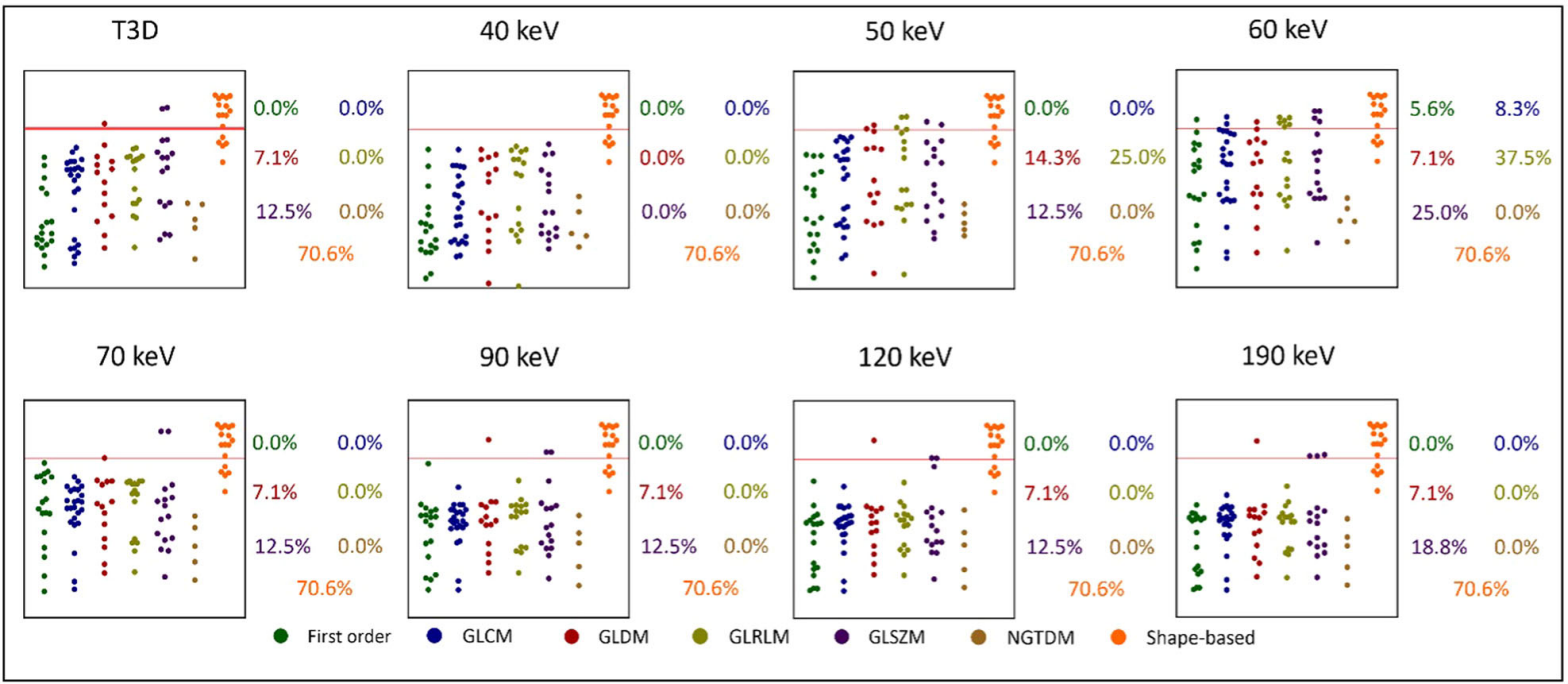
Manhattan plots of radiomic features and their respective ICC values between EID-CT and PCD-CT reconstructions. Manhattan plots of ICC values are presented for each pairwise comparison with EID-CT. The *y*-axis depicts the ICC values, while radiomic features are displayed along the *x*-axis, stratified by radiomic feature classes. The red line indicates an ICC of 0.75 and the proportion of radiomic features with ICC above this is presented for each class. EID-CT, Energy-integrating detector CT; GLCM, Gray level co-occurrence matrix; GLDM, Gray level dependence matrix; GLRLM, Gray level run length matrix; GLSZM, Gray level size zone matrix; ICC, Intraclass correlation coefficient; NGTDM, Neighboring gray-tone difference matrix; PCD-CT, Photon-counting detector CT

A higher proportion of stable features generally led to higher pairwise ICC values, with 11.8% (13/110) of T3D features showing excellent and 12.7% (14/110) demonstrating good reproducibility compared to EID-CT. Similar but slightly more favorable tendencies could be noted for 50 keV (excellent ICC, 18.2% [20/110]; good ICC, 21.8% [24/110]), 60 keV (excellent ICC, 24.5% [27/110]; good ICC, 21.8% [24/110]), and 70 keV (excellent ICC, 13.6% [15/110]; good ICC, 21.8% [24/110]) VMI levels. Median ICC values showed a moderate overall reproducibility of T3D (ICC = 0.50 [0.20–0.59]), 50 keV (ICC = 0.49 [0.22–0.68]), 60 keV (ICC = 0.56 [0.34–0.74]), 70 keV (ICC = 0.50 [0.36–0.62]), and 90 keV (ICC = 0.41 [0.26–0.48]) reconstructions with EID-CT. On the other hand, overall reproducibility for 40 keV (ICC = 0.30 [0.12–0.58]), 120 keV (ICC = 0.39 [0.26–0.47]), and 190 keV (ICC = 0.39 [0.23–0.46]) was poor. Detailed results regarding the reproducibility of radiomic features can be found in Fig. 3 and in Supplementary Table S2.

### Interreader reproducibility

**Fig. 4:**
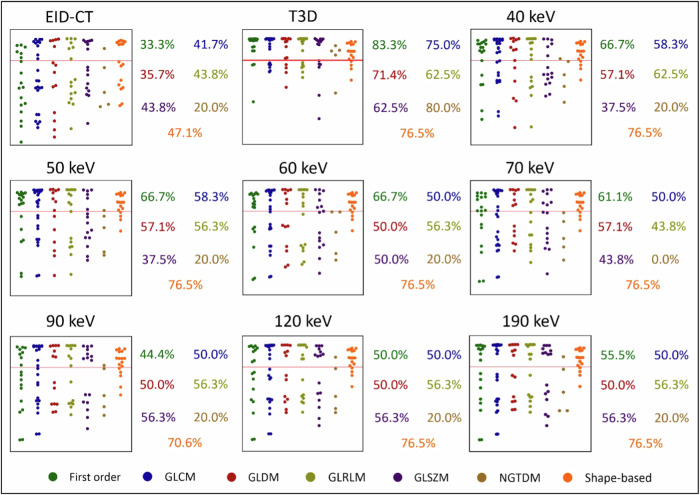
Manhattan plots of radiomic features with corresponding ICC values between the two segmentations performed by different readers. Manhattan plots of ICC values are presented for each pairwise comparison between segmentations. The *y*-axis depicts the ICC values, while radiomic features are displayed along the *z*-axis, stratified by radiomic feature classes. The red line indicates an ICC of 0.75 and the proportion of radiomic features with ICC above this is presented for each class. GLCM, Gray level co-occurrence matrix; GLDM, Gray level dependence matrix; GLRLM, Gray level run length matrix; GLS*Z*M, Gray level size zone matrix; ICC, Intraclass correlation coefficient; NGTDM, Neighboring gray-tone difference matrix

ICC values used to assess reproducibility between segmentations performed by different readers showed that EID-CT-based features had excellent reproducibility for 40.0% (44/110) and good for 10.9% (12/110) of all comparisons, while PCD-CT-based polychromatic reconstruction produced excellent reproducibility for 73.6% (81/110) and good for 20.9% (23/110) of all assessed features. A high proportion of features showed excellent-to-good reproducibility for VMI levels as well, with all the VMI datasets displaying favorable tendencies. Median ICC values showed good overall reproducibility for EID-CT (ICC = 0.61 [0.37–0.93]), and excellent for all PCD-CT reconstructions (highest ICC = 0.95 [0.73–0.99] scored by the polychromatic T3D). A detailed overview of all ICC values resulting from different segmentations of readers is displayed in Fig. 4 and in Supplementary Table S3.

## Discussion

This study compared the radiomic features of myocardial tissue in patients undergoing CCTA with both EID-CT and PCD-CT, utilizing similar acquisition and reconstruction protocols within a brief timeframe. The most important findings are that polychromatic T3D and VMI reconstructions at 60 keV and 70 keV showed the highest proportion of stable features compared to EID-CT. While a considerable percentage of features (39−96%) demonstrated stability in other pairwise comparisons as well, the overall reproducibility of features was moderate even in the most favorable comparisons. On the other hand, the reproducibility of features between segmentations performed by different readers may be superior with the PCD-CT technology (highest ICC = 0.95 [0.73–0.99] by polychromatic T3D) compared to EID-CT (ICC = 0.61 [0.37–0.93]), as all VMI levels and polychromatic T3D PCD-CT reconstructions outperformed the EID-CT series in this aspect.

Cardiac magnetic resonance is still considered the reference standard method for myocardial characterization due to its high soft-tissue contrast and favorable signal-to-noise ratio [24, 25]. The recently introduced PCD-CT system, however, provides improved contrast-to-noise ratio and soft-tissue differentiation over previous EID-CT systems by weighting x-ray quanta equally, regardless of their energy level [26]. Moreover, previous dual-energy CT-based spectral reconstructions have had limited applicability in cardiac imaging due to inherent limitations in temporal resolution. With PCD-CT, spectral data is available in every scan without compromising temporal resolution and does not require specific dual-energy acquisition protocols. The technological advances of PCD-CT, in conjunction with the enhanced spatial resolution of the detector, facilitate the acquisition of a greater number of image details compared to conventional EID-CT.

The potential benefits of the PCD-CT technology may extend to radiomics, where it could offer a more comprehensive representation of encoded information, ultimately leading to differences in texture analysis profiles. The question of whether more detailed radiomic profiles with PCD-CT offer superior predictive models for myocardial pathologies remains unanswered and further research is warranted [20]. These advances promise more reproducible measurements, such as quantitative HU accuracy, when combined with PCD-CT’s ability to eliminate electronic noise [27]. Our findings seem to support this view as EID-CT-based segmentations showed only good reproducibility between readers, while all PCD-CT segmentations demonstrated excellent reproducibility, with approximately 95% for T3D comparisons, producing excellent-to-good ICC values (0.62–1.00).

Myocardial radiomics from CCTA images has focused on identifying objective and quantitative myocardial texture metrics that distinguish healthy from diseased tissue. Previous investigations on EID-CT technology have shown favorable diagnostic performance for first-order and textural features for various purposes. Special attention has been given to characterizing myocardial scar and fibrosis: studies demonstrated the feasibility of different radiomic features for distinguishing health from acutely and chronically infarcted myocardium in CCTA. Interestingly, radiomic texture analysis was also able to identify different patterns of structural micro-architectural remodeling characterizing patients with recurrent ventricular tachycardia [12, 13, 28–30]. The interscanner stability of radiomic features in our study may suggest that these objective textural parameters may also be replicated using specific PCD-CT reconstructions. Polychromatic T3D and VMI reconstructions at 60–70 keV seem to produce radiomic features with considerably high stability compared to EID-CT, potentially proposing the interchangeability of features between scanners. Nonetheless, the moderate reproducibility of features between scanners highlights that previously described EID-CT-based parameters cannot be directly translated into PCD-CT datasets and further investigations must address the unique predictive power of these series too before considering a direct translation of previous results to PCD technology. In a recent publication reporting results in 30 patients [31], authors discovered that a specific set of radiomic features indicating texture changes in the left ventricular myocardium correlated with the severity of coronary artery calcification, estimated by the Agatston score. Considering that the PCD technology may encode different, potentially more complex textural information, similar efforts are warranted to reproduce constellations that had previously been described on EID-CT.

At the advent of photon-counting technology, evidence regarding the reproducibility of PCD-CT-based radiomic features has been scarce in the literature. Ayx et al [32] recently assessed the comparative feature properties of 50 patients (25 patients scanned on PCD-CT *versus* 25 other patients on EID-CT) and found that more than 15% of original radiomic features differed between EID-CT and polychromatic PCD-CT series. Besides extending the head-to-head comparison to different VMI levels as well, the current study suggests that feature stability is, in fact, higher when assessed within the same patient.

The intraindividual and interscanner comparison of radiomic features represents a novelty in the field and may pave the way for further studies with larger populations employing similar designs that could lead to inter-scanner standardization. This becomes increasingly necessary with the continuously growing number of PCD-CT scanners available. Additionally, Wolf et al [33] have previously examined reproducibility across various VMI levels and reported patterns of escalating reproducibility with increasing VMI levels. While these findings align with the currently reported tendencies, the proportion of stable features reported here (95–98%) is considerably higher than that reported by these authors (77–89%). A possible explanation may lie in the study design, as the stability of features was previously assessed only for a single slice of the myocardium, whereas the current study assessed the myocardium as a whole, potentially increasing robustness.

The definition of stability concerning radiomic features is currently not universally accepted. In fact, stability has been previously delineated using both an ICC-derived approach and a significance-based methodology [33, 34]. To ensure a more thorough analysis, we chose to incorporate both methods to elucidate differences between the scanners. The variance in definitions observed across various studies emphasizes the importance of standardization efforts in the field of radiomics.

This study has limitations that merit consideration. First, the number of patients included in this study can be considered limited, although is within the average number of patients for many radiomics studies in the literature. Nevertheless, the uniqueness of this study lies in the enrollment of patients who underwent CCTA with two separate scanners in a short timeframe, providing the best potential for scanner-dependent feature comparison. Furthermore, larger trials that entail exposing patients to two separate CT scans are seldom approved due to radiation safety concerns. Second, while reconstruction parameters were meticulously matched during post-processing, the slight variation in tube voltage settings between PCD-CT and EID-CT acquisitions can be viewed as a potential confounding factor. This could reduce the presented reproducibility of features, but the currently proposed trends are likely to persist in a study that employs matching kV levels too. Third, while the advanced technological features of PCD-CT have the potential to enhance diagnostic accuracy in distinguishing different myocardial pathologies, no such analysis has been conducted currently owing to the low number of scans with pathologies confirmed by reference standard examinations. Additionally, while our study focused on analyzing original radiomic features, examining higher-order features could provide a more comprehensive comparison.

The present study was designed to assess overall trends and may serve as the basis for future endeavors. Lastly, PCD-CT technology has recently enabled the acquisition of ultrahigh-resolution CCTA, which was not yet available at our institution at the time of this current study. It is, however, a topic of interest to explore the effect of an increased spatial resolution on texture features. In this intraindividual study design, we concluded that the majority of original radiomic features remain stable between EID-CT and PCD-CT polychromatic reconstructions (T3D) and certain VMI levels (60 keV and 70 keV). This relative stability, however, does not necessarily translate to high correlation coefficients, warranting the need for standardization and wide-scale validation of PCD-CT-based radiomics before routine implementation in both research and clinical settings. On the other hand, the technological improvements linked with the novel PCD-CT technology may pave the way to a higher reproducibility between readers, ultimately expediting this process.

### Supplementary information

**Additional file 1: Supplementary Table 1.** Radiomic features with significant differences between EID-CT all PCD-CT reconstructions. **Supplementary Table 2.** Two-way mixed intraclass correlation coefficient (ICC) showing reproducibility of original radiomics features between EID-CT and all PCD-CT reconstructions. **Supplementary Table 3.** Two-way mixed intraclass correlation coefficient (ICC) showing inter-reader reproducibility of original radiomics features between the two segmentations performed by different readers.

## Data Availability

The datasets used and/or analyzed during the current study are available from the corresponding author upon reasonable request.

## Abbreviations

CCTA: Coronary computed tomography angiography
EID-CT: Energy-integrating detector computed tomography
GLCM: Gray level co-occurrence matrix
GLDM: Gray level dependence matrix
GLRLM: Gray level run length matrix
GLSZM: Gray level size zone matrix
ICC: Intraclass correlation coefficient
NGTDM: Neighboring gray-tone difference matrix
PCD-CT: Photon-counting detector computed tomography
VMI: Virtual monoenergetic images
