# Supplementary material for: Intraindividual reproducibility of myocardial radiomic features between energy-integrating detector and photon-counting detector CT angiography
Source: Eur Radiol Exp. 2024 Aug 28;8:101. doi: 10.1186/s41747-024-00493-7 (PMC11358367; doi:10.1186/s41747-024-00493-7)
Supplement: Supplementary file 1 — Additional file 1: Supplementary Table 1. Radiomic features with significant differences between EID-CT all PCD-CT reconstructions. Supplementary Table 2. Two-way mixed intraclass correlation coefficient (ICC) showing reproducibility of original radiomics features between EID-CT and all PCD-CT reconstructions. Supplementary Table 3. Two-way mixed intraclass correlation coefficient (ICC) showing inter-reader reproducibility of original radiomics features between the two segmentations performed by different readers. [file 41747_2024_493_MOESM1_ESM.pdf]

Intraindividual reproducibility of myocardial radiomic features between energy-integrating detector and photon-counting detector CT angiography
ELECTRONIC SUPPLEMENTARY MATERIAL

Supplemental table 1. Radiomic features with significant differences between EID-CT all PCD-CT reconstructions.

EID-CT, energy-integrating detector CT; PCD-CT, photon-counting detector CT.

40 keV

Feature	*R^2*	*p*
original_firstorder_Mean	0.749	1.05e-09
original_firstorder_Median	0.725	2.49e-09
original_firstorder_RootMeanSquared	0.710	4.16e-09
original_glcm_Imc2	0.679	1.82e-08
original_ngtdm_Coarseness	0.673	1.99e-08
original_glcm_Correlation	0.658	3.63e-08
original_firstorder_90Percentile	0.649	4.82e-08
original_glszm_ZoneEntropy	0.629	9.66e-08
original_gldm_DependenceEntropy	0.629	9.66e-08
original_glrlm_GrayLevelNonUniformity	0.601	3.16e-07
original_grlm_RunEntropy	0.593	3.98e-07
original_glszm_GrayLevelNonUniformityNormalized	0.567	1.07e-06
original_glcm_Imc1	0.562	1.22e-06
original_glcm_SumEntropy	0.531	3.62e-06
original_firstorder_Entropy	0.504	8.94e-06
original_glrlm_GrayLevelNonUniformityNormalized	0.501	9.62e-06
original_gldm_GrayLevelNonUniformity	0.499	9.62e-06
original_firstorder_Uniformity	0.491	1.18e-05
original_firstorder_Energy	0.418	1.20e-04

original_glcm_JointEntropy	0.414	1.26e-04
original_ngtdm_Busyness	0.395	2.14e-04
original_firstorder_MeanAbsoluteDeviation	0.389	2.42e-04
original_firstorder_TotalEnergy	0.381	2.88e-04
original_glcm_MCC	0.380	2.88e-04
original_firstorder_InterquartileRange	0.370	3.54e-04
original_firstorder_RobustMeanAbsoluteDeviation	0.370	3.54e-04
original_firstorder_10Percentile	0.358	4.67e-04
original_glcm_JointEnergy	0.347	6.13e-04
original_glcm_MaximumProbability	0.344	6.38e-04
original_glszm_GrayLevelVariance	0.342	6.51e-04
original_glszm_SmallAreaLowGrayLevelEmphasis	0.292	2.35e-03
original_glszm_GrayLevelNonUniformity	0.288	2.49e-03
original_glszm_LowGrayLevelZoneEmphasis	0.288	2.49e-03
original_glrlm_ShortRunLowGrayLevelEmphasis	0.281	2.87e-03
original_glrlm_LowGrayLevelRunEmphasis	0.275	3.23e-03
original_glrlm_GrayLevelVariance	0.258	4.70e-03
original_gldm_GrayLevelVariance	0.256	4.70e-03
original_firstorder_Variance	0.256	4.70e-03
original_glcm_DifferenceVariance	0.254	4.83e-03
original_glcm_SumSquares	0.243	6.24e-03
original_glcm_ClusterTendency	0.240	6.46e-03
original_glrlm_RunLengthNonUniformity	0.239	6.46e-03
original_gldm_LowGrayLevelEmphasis	0.232	7.49e-03
original_glcm_Contrast	0.230	7.72e-03
original_glcm_Idmn	0.228	7.91e-03
original_glcm_DifferenceEntropy	0.220	9.35e-03

original_glcm_DifferenceAverage	0.210	1.14e-02
original_glszm_ZoneVariance	0.209	1.14e-02
original_glszm_LargeAreaEmphasis	0.209	1.14e-02
original_glcm_InverseVariance	0.196	1.51e-02
original_gldm_SmallDependenceEmphasis	0.193	1.59e-02
original_glszm_ZonePercentage	0.190	1.66e-02
original_gldm_SmallDependenceLowGrayLevelEmphasis	0.182	1.95e-02
original_gicm_Id	0.181	1.95e-02
original_glszm_SmallAreaEmphasis	0.180	1.99e-02
original_glcm_Idm	0.179	1.998-02
original_glrlm_RunLengthNonUniformityNormalized	0.178	2.01e-02
original_glszm_LargeAreaLowGrayLevelEmphasis	0.173	2.21e-02
original_glrlm_ShortRunEmphasis	0.171	2.27e-02
original_glrlm_RunPercentage	0.168	2.38e-02
original_glcm_Idn	0.166	2.46e-02
original_glszm_SizeZoneNonUniformityNormalized	0.164	2.55e-02
original_gldm_DependenceNonUniformity	0.158	2.866-02
original_gldm_LargeDependenceEmphasis	0.156	2.936-02
original_gldm_DependenceNonUniformityNormalized	0.151	3.23e-02
original_firstorder_Maximum	0.140	4.12e-02
original_girim_LongRunEmphasis	0.137	4.338-02

50 keV

Feature	***R^2***	***p***
original_firstorder_Mean	0.705	1.71e-08
original_firstorder_Median	0.678	3.93e-08
original_ngtdm_Coarseness	0.639	1.86e-07

original_firstorder_RootMeanSquared	0.613	4.55e-07
original_firstorder_90Percentile	0.524	1.32e-05
original_glrlm_GrayLevelNonUniformity	0.486	4.23e-05
original_glszm_ZoneEntropy	0.447	1.31e-04
original_glcm_Imc2	0.421	2.55e-04
original_glcm_Correlation	0.404	3.84e-04
original_gldm_GrayLevelNonUniformity	0.371	9.00e-04
original_glcm_Imc1	0.361	1.06e-03
original_qldm_DependenceEntropy	0.356	1.14e-03
original_firstorder_10Percentile	0.349	1.27e-03
original_glrlm_RunLengthNonUniformity	0.338	1.59e-03
original_glszm_GrayLevelNonUniformity	0.333	1.67e-03
original_glrlm_RunEntropy	0.329	1.74e-03
original_firstorder_TotalEnergy	0.303	3.22e-03
original_ngtdm_Busyness	0.294	3.89e-03
original_gldm_DependenceNonUniformity	0.276	5.75e-03
original_glszm_GrayLevelNonUniformityNormalized	0.271	6.21e-03
original_firstorder_Energy	0.269	6.21e-03
original_glcm_SumEntropy	0.232	1.46e-02
original_firstorder_Entropy	0.209	2.42e-02
original_glszm_GrayLevelVariance	0.205	2.54e-02
original_firstorder_MeanAbsoluteDeviation	0.198	2.90e-02
original_glcm_MCC	0.183	3.93e-02
original_glrlm_GrayLevelNonUniformityNormalized	0.177	4.14e-02
original_glrlm_GrayLevelVariance	0.175	4.14e-02
original_gldm_GrayLevelVariance	0.175	4.14e-02
original_firstorder_Variance	0.174	4.14e-02

original_firstorder_RobustMeanAbsoluteDeviation	0.172	4.16e-02
original_firstorder_Uniformity	0.171	4.166-02
original_glcm_ClusterTendency	0.17	4.16e-02
original_firstorder_InterquartileRange	0.169	4.16e-02
original_glcm_SumSquares	0.164	4.45e-02

60 keV

Feature	***R^2***	***p***
original_ngtdm_Coarseness	0.594	2.95e-06
original_firstorder_Mean	0.586	2.95e-06
original_firstorder_Median	0.553	7.40e-06
original_glrlm_RunLengthNonUniformity	0.415	6.20e-04
original_firstorder_RootMeanSquared	0.386	1.17e-03
original_gldm_DependenceNonUniformity	0.367	1.57e-03
original_glrlm_GrayLevelNonUniformity	0.363	1.57e-03
original_glszm_GrayLevelNonUniformity	0.348	2.12e-03
original_firstorder_10Percentile	0.289	8.72e-03
original_firstorder_90Percentile	0.263	1.53e-02
original_gldm_GrayLevelNonUniformity	0.232	2.96e-02

70 keV

Feature	***R^2***	***p***
original_ngtdm_Coarseness	0.549	2.65e-05
original_glrlm_RunLengthNonUniformity	0.511	5.31e-05
original_gldm_DependenceNonUniformity	0.47	1.448-04
original_glszm_GrayLevelNonUniformity	0.394	1.16e-03
original_firstorder_10Percentile	0.258	3.428-02

original_glszm_SizeZoneNonUniformity	0.241	3.50e-02
original_glrlm_GrayLevelNonUniformity	0.241	3.50e-02
original_firstorder_Median	0.238	3.50e-02
original_firstorder_Mean	0.23	3.79e-02

90 keV

Feature	***R^2***	***p***
original_firstorder Mean	0.599	3.38e-06
original_glrlm_RunLengthNonUniformity	0.567	6.55e-06
original_gidm_Dependence NonUniformity	0.522	2.33e-05
original_firstorder_Energy	0.509	2.33e-05
original_ngtdm_Coarseness	0.505	2.33e-05
original_firstorder_Median	0.503	2.33e-05
original_firstorder_90Percentile	0.453	1.07e-04
original_glszm_GrayLevelNonUniformity	0.398	5.07e-04
original_firstorder_RootMeanSquared	0.334	2.75e-03
original_glszm_SizeZoneNonUniformityNormalized	0.313	4.06e-03
original_girlm_GrayLevelNonUniformityNormalized	0.31	4.06e-03
original_firstorder_Uniformity	0.303	4.06e-03
original_glszm_GrayLevelNonUniformityNormalized	0.301	4.06e-03
original_glcm_JointEntropy	0.298	4.06e-03
original_glszm_SizeZoneNonUniformity	0.295	4.06e-03
original_firstorder_Entropy	0.295	4.06e-03
original_glcm_SumEntropy	0.292	4.06e-03
original_glszm_SmallAreaEmphasis	0.292	4.06e-03
original_glcm_DifferenceEntropy	0.285	4.46e-03
original_firstorder_RobustMeanAbsoluteDeviation	0.284	4.46e-03

original_firstorder_InterquartileRange	0.28	4.46e-03
original_glcm_MaximumProbability	0.28	4.46e-03
original_glcm_JointEnergy.	0.279	4.46e-03
original_gldm_DependenceEntropy	0.272	5.04e-03
original_glcm_Id	0.263	5.93e-03
original_glcm_Idm	0.262	5.93e-03
original_glcm_DifferenceAverage	0.259	6.16e-03
original_gldm_SmallDependenceEmphasis	0.258	6.16e-03
original_firstorder_MeanAbsoluteDeviation	0.254	6.53e-03
original_glrlm_RunEntropy	0.252	6.53e-03
original_glrlm_RunLengthNonUniformit‹Normalized	0.25	6.53e-03
original_glszm_ZonePercentage	0.249	6.53e-03
original_grlm_ShortRunEmphasis	0.249	6.53e-03
original_glrlm_RunPercentage	0.242	7.49e-03
original_gldm_Large DependenceEmphasis	0.232	9.33e-03
original_glcm_Contrast	0.225	1.07e-02
original_glrlm_LongRunEmphasis	0.22	1.16e-02
original_glrlm_RunVariance	0.215	1.26e-02
original_glcm_DifferenceVariance	0.205	1.57e-02
original_gldm_DependenceVariance	0.198	1.75e-02
original_glcm_InverseVariance	0.198	1.75e-02
original_gldm_DependenceNonUniformityNormalized	0.186	2.28e-02
original_firstorder_TotalEnergy	0.173	2.97e-02
original_gldm_LargeDependenceLowGrayLevelEmphasis	0.16	3.91e-02
original_glrlm_LongRunLowGrayLevelEmphasis	0.159	3.92e-02
original_glrlm_GrayLevelVariance	0.155	4.16e-02
original_glrlm_GrayLevelNonUniformity	0.154	4.17e-02

120 keV

Feature	*R^2*	*p*
original_firstorder_Mean	0.818	4.41e-12
original_firstorder_Median	0.809	4.88e-12
original_firstorder_90Percentile	0.66	6.60e-08
original_firstorder_Energy	0.618	3.69e-07
original_firstorder_RootMeanSquared	0.599	6.76e-07
original_glrlm_RunLengthNonUniformity	0.584	1.08e-06
original_gldm_DependenceNonUniformity	0.536	6.13e-06
original_ngtdm_Coarseness	0.498	2.09e-05
original_glszm_GrayLevelNonUniformity	0.417	2.59e-04
original_glrlm_GrayLevelNonUniformityNormalized	0.361	1.18e-03
original_glszm_SizeZoneNonUniformityNormalized	0.357	1.18e-03
original_firstorder_Entropy	0.349	1.21e-03
original_firstorder_Uniformity	0.348	1.21e-03
original_glszm_GrayLevelNonUniformityNormalized	0.348	1.21e-03
original_glcm_JointEntropy	0.344	1.26e-03
original_glszm_SmallAreaEmphasis	0.34	1.28e-03
original_gicm_SumEntropy	0.338	1.28e-03
original_glcm_Difference Entropy	0.333	1.41e-03
original_glcm_MaximumProbability	0.322	1.62e-03
original_firstorder_RobustMeanAbsoluteDeviation	0.322	1.62e-03
original_gldm_DependenceEntropy	0.322	1.62e-03
original_glcm_JointEnergy	0.316	1.76e-03
original_firstorder_InterquartileRange	0.314	1.76e-03

original_glrlm_RunEntropy	0.313	1.76e-03
original_glszm_SizeZoneNonUniformity	0.311	1.76e-03
original_glcm_Idm	0.309	1.76e-03
original_glcm_Id	0,309	1.76e-03
original_firstorder_TotalEnergy	0,302	1.90e-03
original_firstorder_MeanAbsoluteDeviation	0.302	1.90e-03
original_glcm_DifferenceAverage	0.302	1.90e-03
original_glrlm_RunLengthNonUniformityNormalized	0,297	2.06e-03
original_gldm_SmallDependenceEmphasis	0.296	2.06e-03
original_glrlm_ShortRunEmphasis	0.294	2.11e-03
original_glrlm_RunPercentage	0.286	2.49e-03
original_glszm_ZonePercentage	0.282	2.69e-03
original_gldm_LargeDependenceEmphasis	0.273	3.31e-03
original_glcm_Contrast	0.258	4.64e-03
original_glrlm_LongRunEmphasis	0.255	4.93e-03
original_glrlm_RunVariance	0.249	5.53e-03
original_glcm_DifferenceVariance	0.237	7.21e-03
original_gldm_DependenceVariance	0.234	7.55e-03
original_glcm_InverseVariance	0.222	9.83e-03
original_gldm_DependenceNonUniformityNormalized	0.22	9.91e-03
original_glrlm_GrayLevelVariance	0.196	1.72e-02
original_gldm_LargeDependenceLowGrayLevelEmphasis	0.167	3.25e-02
original_glrlm_LongRunLowGrayLevelEmphasis	0.164	3.39e-02

190 keV

Feature	***R^2***	***p***

original_firstorder_Median	0.842	2.19e-13
original_firstorder_Mean	0.841	2.19e-13
original_firstorder_90Percentile	0.72	2.28e-09
original_firstorder_RootMeanSquared	0.676	2.10e-08
original_firstorder_Energy	0.655	5.04e-08
original_glrlm_RunLengthNonUniformity	0.591	8.01e-07
original_gldm_DependenceNonUniformity	0.542	4.90e-06
original_ngtdm_Coarseness	0.515	1.14e-05
original_glszm_GrayLevelNonUniformity	0.447	1.03e-04
original_glszm_SizeZoneNonUniformityNormalized	0.361	1.18e-03
original_firstorder_TotalEnergy	0.356	1.19e-03
original_glrlm_GrayLevelNonUniformityNormalized	0.353	1.19e-03
original_glcm_DifferenceEntropy	0.351	1.19e-03
original_glszm_SmallAreamphasis	0.345	1.31e-03
original_glcm_JointEntropy	0.339	1.31e-03
original_firstorder_Uniformity	0.339	1.31e-03
original_firstorder_Entropy	0.338	1.31e-03
original_glszm_GrayLevelNonUniformityNormalized	0.331	1.44e-03
original_glcm_Idm	0.328	1.44e-03
original_glcm_Id	0.328	1.44e-03
original_glcm_DifferenceAverage	0.319	1.62e-03
original_glrlm_RunLengthNonUniformityNormalized	0.317	1.62e-03
original_glszm_SizeZoneNonUniformity	0.316	1.62e-03
original_glcm_JointEnergy	0.316	1.62e-03
original_glcm_SumEntropy	0.314	1.62e-03
original_glrlm_ShortRunEmphasis	0.314	1.62e-03
original_gldm_SmallDependenceEmphasis	0.311	1.66e-03

original_glcm_MaximumProbability	0.306	1.80e-03
original_glrlm_RunPercentage	0.305	1.80e-03
original_firstorder_RobustMeanAbsoluteDeviation	0,303	1.86e-03
original_glszm_ZonePercentage	0.297	2.08e-03
original_firstorder_InterquartileRange	0.296	2.08e-03
original_gldm_LargeDependenceEmphasis	206	2.30e-03
original_firstorder_MeanAbsolute Deviation	0.291	2,37e-03
original_glcm_Contrast	0.288	3.62e-03
original_glrlm_LongRunEmphasis	0.27	3.68e-03
original_glrlm_RunVariance	0.269	4 218-03
original_gldm_DependenceEntropy	0.262	4.34e-03
original_glrlm_RunEntropy	0.259	4.34e-03
original_gldm_DependenceVariance	0.259	5.22e-03
original_gicm_DifferenceVariance	0.25	5.33e-03
original_gldm_DependenceNonUniformityNormalized	0.248	7.07e-03
original_glcm_InverseVariance	0.235	8.57e-03
original_firstorder_10Percentile	0.226	9.49e-03
original_glrlm_GrayLevelVariance	0.221	1.85e-02
original_gldm_LargeDependenceLowGrayLevelEmphasis	0.192	3.38e-02
original_glrlm_LongRunLowGrayLevelEmphasis	0.165	3.56e-02
original_glrlm_GrayLevelNonUniformity	0.161	3.90e-02

T3D

Feature	***R^2***	***p***
original_grlm_GrayLevelNonUniformity	0.346	1.75e-02
original_ngtdm_Coarseness	0.309	2.38e-02
original_gldm_GrayLevelNonUniformity	0.288	2.72e-02

original_firstorder_Median	0.255	4.60e-02

Supplemental Table 2. Two-way mixed intraclass correlation coefficient (ICC) showing reproducibility of original radiomics features between EID-CT and all PCD-CT reconstructions.

EID-CT, energy-integrating detector CT; PCD-CT, photon-counting detector CT, IQR, Interquartile range.

Feature	***T3D***	***40 keV***	***50 keV***	***60 keV***	***70 keV***	***90 keV***	***120 keV***	***190 keV***
firstorder_10Percentile	0,37	0,14	0,22	0,34	0,43	0,72	0,62	0,39
firstorder_90Percentile	0,08	0,06	0,13	0,33	0,67	0,24	0,10	0,07
firstorder_Energy	0,06	0,04	0,13	0,35	0,31	0,16	0,10	0,09
firstorder_Entropy	0,46	0,24	0,48	0,73	0,64	0,43	0,39	0,39
firstorder_InterquartileRange	0,10	0,19	0,40	0,66	0,65	0,41	0,37	0,38
firstorder_Kurtosis	-0,07	-0,11	-0,13	-0,09	-0,04	-0,03	-0,03	-0,04
firstorder_Maximum	0,19	0,41	0,50	0,50	0,45	0,35	0,31	0,31
firstorder_Mean	0,09	0,01	0,03	0,07	0,22	0,03	-0,02	-0,02
firstorder_MeanAbsoluteDeviation	0,17	0,17	0,36	0,62	0,66	0,46	0,41	0,42
firstorder_Median	0,13	0,01	0,03	0,06	0,16	0,04	-0,02	-0,03
firstorder_Minimum	0,53	0,63	0,60	0,53	0,49	0,48	0,48	0,47
firstorder_Range	0,40	0,53	0,60	0,56	0,51	0,45	0,42	0,42
firstorder_RobustMeanAbsoluteDeviation	0,13	0,18	0,39	0,65	0,64	0,41	0,37	0,38
firstorder_RootMeanSquared	0,06	0,03	0,07	0,17	0,42	0,26	0,13	0,10
firstorder_Skewness	0,00	-0,14	-0,04	0,02	0,05	0,06	0,06	0,06
firstorder_TotalEnergy	0,20	0,09	0,23	0,52	0,73	0,40	0,25	0,22
firstorder_Uniformity	0,58	0,28	0,60	0,80	0,61	0,40	0,36	0,38
firstorder_Variance	0,04	0,08	0,19	0,36	0,42	0,38	0,37	0,40
glcm_Autocorrelation	0,51	0,63	0,63	0,55	0,49	0,48	0,47	0,47
glcm_ClusterProminence	-0,05	-0,01	-0,02	-0,03	-0,03	-0,03	-0,04	-0,04
glcm_ClusterShade	-0,01	-0,01	0,00	0,01	0,02	0,02	0,02	0,02

glcm_ClusterTendency	0,01	0,07	0,17	0,32	0,40	0,36	0,37	0,40
glcm_Contrast	0,48	0,28	0,52	0,60	0,46	0,34	0,30	0,29
glcm_Correlation	0,06	0,09	0,18	0,32	0,37	0,38	0,41	0,45
glcm_DifferenceAverage	0,51	0,41	0,62	0,67	0,53	0,40	0,36	0,35
glcm_DifferenceEntropy	0,57	0,47	0,68	0,72	0,57	0,42	0,37	0,36
glcm_DifferenceVariance	0,52	0,23	0,48	0,59	0,45	0,33	0,30	0,29
glcm_Id	0,56	0,52	0,71	0,73	0,59	0,45	0,40	0,39
glcm_Idm	0,56	0,53	0,71	0,74	0,59	0,45	0,40	0,38
glcm_Idmn	0,39	0,11	0,21	0,33	0,36	0,35	0,41	0,42
glcm_Idn	0,38	0,18	0,29	0,39	0,45	0,44	0,46	0,44
glcm_Imc1	0,04	0,17	0,28	0,43	0,49	0,58	0,59	0,53
glcm_Imc2	0,09	0,08	0,17	0,32	0,36	0,34	0,35	0,39
glcm_InverseVariance	0,47	0,43	0,59	0,63	0,46	0,26	0,19	0,16
glcm_JointAverage	0,51	0,53	0,57	0,54	0,50	0,48	0,47	0,46
glcm_JointEnergy	0,64	0,35	0,70	0,82	0,57	0,38	0,35	0,35
glcm_JointEntropy	0,55	0,31	0,57	0,74	0,62	0,43	0,39	0,39
glcm_MaximumProbability	0,61	0,36	0,68	0,78	0,56	0,36	0,35	0,38
glcm_MCC	0,27	0,06	0,11	0,16	0,19	0,25	0,26	0,27
glcm_SumAverage	0,51	0,53	0,57	0,54	0,50	0,48	0,47	0,46
glcm_SumEntropy	0,45	0,22	0,46	0,72	0,64	0,43	0,39	0,41
glcm_SumSquares	0,04	0,08	0,19	0,36	0,43	0,38	0,37	0,40
gldm_DependenceEntropy	0,23	0,15	0,32	0,60	0,62	0,40	0,37	0,42
gldm_DependenceNonUniformity	0,66	0,57	0,45	0,36	0,27	0,24	0,23	0,23
gldm_DependenceNonUniformityNormalized	0,49	0,48	0,64	0,69	0,60	0,49	0,46	0,44
gldm_DependenceVariance	0,56	0,60	0,74	0,75	0,62	0,49	0,46	0,44

gldm_GrayLevelNonUniformity	0,43	0,22	0,36	0,54	0,76	0,86	0,86	0,85
gldm_GrayLevelVariance	0,04	0,08	0,19	0,36	0,43	0,38	0,37	0,40
gldm_HighGrayLevelEmphasis	0,51	0,63	0,63	0,55	0,49	0,48	0,47	0,47
gldm_LargeDependenceEmphasis	0,59	0,58	0,78	0,79	0,61	0,46	0,41	0,39
gldm_LargeDependenceHighGrayLevelEmphasis	0,12	0,23	0,20	0,16	0,12	0,13	0,13	0,12
gldm_LargeDependenceLowGrayLevelEmphasis	0,20	-0,17	-0,11	0,01	0,07	0,07	0,06	0,04
gldm_LowGrayLevelEmphasis	0,30	0,02	0,18	0,28	0,22	0,18	0,18	0,15
gldm_SmallDependenceEmphasis	0,54	0,44	0,64	0,67	0,48	0,36	0,31	0,30
gldm_SmallDependenceHighGrayLevelEmphasis	0,78	0,51	0,76	0,67	0,46	0,35	0,32	0,31
gldm_SmallDependenceLowGrayLevelEmphasis	0,37	0,25	0,37	0,40	0,36	0,38	0,44	0,41
glrlm_GrayLevelNonUniformity	0,39	0,18	0,29	0,41	0,54	0,62	0,61	0,58
glrlm_GrayLevelNonUniformityNormalized	0,58	0,28	0,58	0,80	0,62	0,41	0,36	0,37
glrlm_GrayLevelVariance	0,04	0,08	0,21	0,45	0,56	0,43	0,40	0,42
glrlm_HighGrayLevelRunEmphasis	0,51	0,63	0,64	0,55	0,50	0,48	0,47	0,47
glrlm_LongRunEmphasis	0,63	0,62	0,83	0,82	0,60	0,44	0,39	0,37
glrlm_LongRunHighGrayLevelEmphasis	0,31	0,47	0,39	0,29	0,20	0,19	0,18	0,17
glrlm_LongRunLowGrayLevelEmphasis	0,23	-0,19	-0,11	0,02	0,07	0,07	0,06	0,04
glrlm_LowGrayLevelRunEmphasis	0,31	0,12	0,28	0,34	0,25	0,20	0,21	0,18
glrlm_RunEntropy	0,22	0,15	0,30	0,56	0,60	0,40	0,35	0,40
glrlm_RunLengthNonUniformity	0,59	0,49	0,39	0,32	0,25	0,22	0,21	0,20
glrlm_RunLengthNonUniformityNormalized	0,57	0,55	0,73	0,76	0,61	0,47	0,42	0,40
glrlm_RunPercentage	0,58	0,57	0,75	0,77	0,62	0,47	0,43	0,41
glrlm_RunVariance	0,64	0,62	0,83	0,82	0,60	0,43	0,39	0,37
glrlm_ShortRunEmphasis	0,59	0,57	0,76	0,78	0,62	0,47	0,42	0,40
glrlm_ShortRunHighGrayLevelEmphasis	0,56	0,65	0,68	0,60	0,54	0,51	0,50	0,49

glrlm_ShortRunLowGrayLevelEmphasis	0,33	0,15	0,31	0,35	0,27	0,22	0,24	0,21
glszm_GrayLevelNonUniformity	0,61	0,42	0,37	0,34	0,26	0,26	0,24	0,22
glszm_GrayLevelNonUniformityNormalized	0,69	0,18	0,42	0,72	0,47	0,34	0,32	0,36
glszm_GrayLevelVariance	0,12	0,09	0,21	0,36	0,39	0,35	0,36	0,37
glszm_HighGrayLevelZoneEmphasis	0,52	0,61	0,64	0,56	0,50	0,47	0,46	0,45
glszm_LargeAreaEmphasis	0,58	0,25	0,55	0,85	0,91	0,79	0,76	0,76
glszm_LargeAreaHighGrayLevelEmphasis	0,09	0,11	0,10	0,07	0,04	0,04	0,04	0,04
glszm_LargeAreaLowGrayLevelEmphasis	0,12	0,04	0,14	0,33	0,30	0,25	0,27	0,27
glszm_LowGrayLevelZoneEmphasis	0,29	0,13	0,29	0,33	0,20	0,17	0,20	0,17
glszm_SizeZoneNonUniformity	0,68	0,66	0,68	0,49	0,28	0,21	0,19	0,18
glszm_SizeZoneNonUniformityNormalized	0,87	0,51	0,80	0,79	0,57	0,45	0,41	0,40
glszm_SmallAreaEmphasis	0,88	0,48	0,78	0,81	0,59	0,49	0,44	0,43
glszm_SmallAreaHighGrayLevelEmphasis	0,61	0,60	0,68	0,61	0,51	0,46	0,43	0,42
glszm_SmallAreaLowGrayLevelEmphasis	0,32	0,16	0,33	0,38	0,21	0,22	0,26	0,23
glszm_ZoneEntropy	0,31	0,13	0,24	0,43	0,52	0,62	0,71	0,77
glszm_ZonePercentage	0,50	0,38	0,59	0,61	0,41	0,29	0,26	0,24
glszm_ZoneVariance	0,58	0,25	0,55	0,85	0,91	0,79	0,76	0,76
ngtdm_Busyness	0,21	0,05	0,12	0,27	0,34	0,31	0,31	0,28
ngtdm_Coarseness	0,16	0,11	0,15	0,19	0,23	0,25	0,24	0,23
ngtdm_Complexity	0,31	0,35	0,31	0,20	0,13	0,11	0,09	0,10
ngtdm_Contrast	0,30	0,13	0,25	0,33	0,41	0,41	0,45	0,39
ngtdm_Strength	-0,03	0,26	0,19	0,07	0,03	-0,01	-0,02	-0,01
shape_Compactness1	0,65	0,65	0,65	0,65	0,65	0,65	0,65	0,65
shape_Compactness2	0,57	0,55	0,55	0,55	0,55	0,55	0,55	0,55
shape_Elongation	0,51	0,77	0,77	0,77	0,77	0,77	0,77	0,77

shape_Flatness	0,68	0,86	0,86	0,86	0,86	0,86	0,86	0,86
shape_LeastAxisLength	0,91	0,90	0,90	0,90	0,90	0,90	0,90	0,90
shape_MajorAxisLength	0,96	0,95	0,95	0,95	0,95	0,95	0,95	0,95
shape_Maximum2DDiameterColumn	0,88	0,95	0,95	0,95	0,95	0,95	0,95	0,95
shape_Maximum2DDiameterRow	0,91	0,89	0,89	0,89	0,89	0,89	0,89	0,89
shape_Maximum2DDiameterSlice	0,93	0,95	0,95	0,95	0,95	0,95	0,95	0,95
shape_Maximum3DDiameter	0,94	0,94	0,94	0,94	0,94	0,94	0,94	0,94
shape_MeshVolume	0,94	0,84	0,84	0,84	0,84	0,84	0,84	0,84
shape_MinorAxisLength	0,85	0,94	0,94	0,94	0,94	0,94	0,94	0,94
shape_SphericalDisproportion	0,71	0,70	0,70	0,70	0,70	0,70	0,70	0,70
shape_Sphericity	0,67	0,67	0,67	0,67	0,67	0,67	0,67	0,67
shape_SurfaceArea	0,89	0,83	0,83	0,83	0,83	0,83	0,83	0,83
shape_SurfaceVolumeRatio	0,72	0,67	0,67	0,67	0,67	0,67	0,67	0,67
shape_VoxelVolume	0,94	0,83	0,83	0,83	0,83	0,83	0,83	0,83
Median value	0,50	0,30	0,49	0,56	0,50	0,41	0,39	0,39
IQR 1	0,20	0,12	0,22	0,34	0,36	0,26	0,26	0,23
IQR 3	0,59	0,58	0,68	0,74	0,62	0,48	0,47	0,46

Supplemental Table 3. Two-way mixed intraclass correlation coefficient (ICC) showing inter-reader reproducibility of original radiomics features between the two segmentations performed by different readers.

EID-CT, energy-integrating detector CT; IQR, Interquartile range.

Feature	*EID-CT*	*T3D*	*40 keV*	*50 keV*	*60 keV*	*70 keV*	*90 keV*	*120 keV*	*190 keV*
firstorder_10Percentile	0,74	0,98	0,97	0,96	0,94	0,91	0,90	0,92	0,95
firstorder_90Percentile	0,78	0,99	0,86	0,84	0,83	0,80	0,86	0,96	0,99
firstorder_Energy	0,17	0,90	0,86	0,84	0,79	0,71	0,55	0,50	0,51
firstorder_Entropy	0,83	0,98	0,93	0,93	0,93	0,92	0,96	0,97	0,98
firstorder_InterquartileRange	0,93	1,00	0,95	0,95	0,96	0,96	0,98	0,99	0,99
firstorder_Kurtosis	0,27	0,28	0,09	0,02	-0,03	-0,04	-0,07	-0,07	-0,07
firstorder_Maximum	0,50	0,86	0,48	0,43	0,52	0,60	0,70	0,69	0,67
firstorder_Mean	0,01	0,97	0,93	0,91	0,86	0,76	0,27	0,68	0,89
firstorder_MeanAbsoluteDeviation	0,45	0,99	0,90	0,90	0,90	0,88	0,92	0,94	0,95
firstorder_Median	0,85	0,98	0,98	0,97	0,95	0,92	0,66	0,82	0,94
firstorder_Minimum	0,44	0,71	0,48	0,41	0,34	0,35	0,37	0,38	0,39
firstorder_Range	0,61	0,86	0,58	0,55	0,55	0,56	0,61	0,62	0,61
firstorder_RobustMeanAbsoluteDeviation	0,90	1,00	0,93	0,94	0,95	0,95	0,98	0,99	0,99
firstorder_RootMeanSquared	0,33	0,99	0,90	0,88	0,84	0,75	0,52	0,36	0,34
firstorder_Skewness	-0,16	0,70	0,18	0,05	-0,01	-0,05	-0,07	-0,06	-0,07
firstorder_TotalEnergy	0,25	0,91	0,84	0,83	0,80	0,76	0,78	0,81	0,82
firstorder_Uniformity	0,92	0,98	0,96	0,96	0,96	0,96	0,97	0,98	0,99
firstorder_Variance	0,07	1,00	0,75	0,69	0,57	0,28	0,25	0,23	0,26
glcm_Autocorrelation	0,45	0,70	0,51	0,40	0,30	0,30	0,34	0,36	0,37
glcm_ClusterProminence	0,00	0,88	0,17	0,02	0,00	0,00	0,00	0,00	0,00

glcm_ClusterShade	0,04	0,62	0,13	0,03	0,01	0,00	0,00	0,00	-0,01
glcm_ClusterTendency	0,06	1,00	0,73	0,68	0,56	0,37	0,24	0,22	0,26
glcm_Contrast	0,42	1,00	0,94	0,96	0,96	0,95	0,94	0,94	0,94
glcm_Correlation	0,45	0,94	0,84	0,81	0,74	0,69	0,67	0,62	0,60
glcm_DifferenceAverage	0,95	1,00	0,98	0,99	0,99	0,99	1,00	1,00	1,00
glcm_DifferenceEntropy	0,96	1,00	0,97	0,98	0,98	0,98	0,99	0,99	0,99
glcm_DifferenceVariance	0,14	1,00	0,85	0,88	0,88	0,80	0,74	0,73	0,24
glcm_Id	0,99	1,00	0,99	0,99	0,99	0,99	1,00	1,00	1,00
glcm_Idm	0,99	1,00	0,99	0,99	0,99	0,99	1,00	1,00	1,00
glcm_Idmn	0,52	0,67	0,61	0,59	0,57	0,46	0,55	0,65	0,67
glcm_Idn	0,53	0,72	0,58	0,56	0,55	0,52	0,58	0,63	0,64
glcm_Imc1	0,77	0,99	0,89	0,86	0,80	0,76	0,83	0,89	0,93
glcm_Imc2	0,64	0,91	0,88	0,84	0,77	0,72	0,77	0,84	0,90
glcm_InverseVariance	0,95	1,00	0,99	0,99	0,99	0,99	0,99	0,99	0,99
glcm_JointAverage	0,44	0,71	0,52	0,44	0,35	0,36	0,37	0,39	0,39
glcm_JointEnergy	0,96	0,99	0,98	0,98	0,98	0,98	0,99	0,99	0,99
glcm_JointEntropy	0,92	0,99	0,96	0,96	0,96	0,96	0,98	0,99	0,99
glcm_MaximumProbability	0,99	0,99	0,99	0,99	0,99	0,99	0,99	0,99	1,00
glcm_MCC	0,15	0,63	0,50	0,37	0,25	0,31	0,41	0,38	0,36
glcm_SumAverage	0,44	0,71	0,52	0,44	0,35	0,36	0,37	0,39	0,39
glcm_SumEntropy	0,81	0,98	0,94	0,94	0,93	0,92	0,96	0,98	0,98
glcm_SumSquares	0,07	1,00	0,75	0,70	0,60	0,43	0,29	0,29	0,32
gldm_DependenceEntropy	0,62	0,97	0,87	0,86	0,84	0,82	0,87	0,91	0,94
gldm_DependenceNonUniformity	0,91	0,91	0,90	0,89	0,89	0,88	0,88	0,88	0,88
gldm_DependenceNonUniformityNormalized	0,99	1,00	1,00	1,00	1,00	1,00	1,00	1,00	1,00

gldm_DependenceVariance	0,70	1,00	0,99	1,00	1,00	1,00	1,00	1,00	1,00
gldm_GrayLevelNonUniformity	0,97	0,96	0,96	0,96	0,96	0,95	0,94	0,93	0,93
gldm_GrayLevelVariance	0,07	1,00	0,75	0,70	0,57	0,39	0,25	0,24	0,27
gldm_HighGrayLevelEmphasis	0,46	0,70	0,52	0,40	0,30	0,30	0,34	0,36	0,37
gldm_LargeDependenceEmphasis	0,98	1,00	1,00	1,00	1,00	1,00	1,00	1,00	1,00
gldm_LargeDependenceHighGrayLevelEmphasis	0,17	0,45	0,32	0,25	0,12	0,83	0,25	0,27	0,27
gldm_LargeDependenceLowGrayLevelEmphasis	-0,03	0,62	-0,01	0,03	0,14	0,38	0,34	0,39	0,36
gldm_LowGrayLevelEmphasis	-0,11	0,67	0,30	0,31	0,35	0,47	0,34	0,42	0,41
gldm_SmallDependenceEmphasis	0,99	1,00	0,98	0,98	0,99	0,99	0,99	0,99	0,99
gldm_SmallDependenceHighGrayLevelEmphasis	0,73	0,78	0,87	0,76	0,59	0,53	0,58	0,61	0,64
gldm_SmallDependenceLowGrayLevelEmphasis	0,47	0,78	0,67	0,54	0,58	0,58	0,52	0,66	0,65
glrlm_GrayLevelNonUniformity	0,95	0,94	0,94	0,93	0,91	0,88	0,85	0,84	0,84
glrlm_GrayLevelNonUniformityNormalized	0,90	0,98	0,95	0,95	0,95	0,94	0,96	0,97	0,98
glrlm_GrayLevelVariance	0,32	0,99	0,76	0,75	0,72	0,63	0,59	0,61	0,66
glrlm_HighGrayLevelRunEmphasis	0,46	0,70	0,52	0,41	0,30	0,30	0,34	0,36	0,37
glrlm_LongRunEmphasis	0,77	1,00	0,99	1,00	1,00	1,00	1,00	1,00	1,00
glrlm_LongRunHighGrayLevelEmphasis	0,26	0,56	0,36	0,26	0,18	0,17	0,22	0,24	0,24
glrlm_LongRunLowGrayLevelEmphasis	-0,01	0,62	0,00	0,04	0,15	0,37	0,30	0,35	0,31
glrlm_LowGrayLevelRunEmphasis	0,28	0,67	0,44	0,35	0,36	0,48	0,34	0,42	0,41
glrlm_RunEntropy	0,54	0,97	0,88	0,85	0,81	0,74	0,77	0,84	0,89
glrlm_RunLengthNonUniformity	0,89	0,86	0,81	0,83	0,84	0,86	0,88	0,89	0,89
glrlm_RunLengthNonUniformityNormalized	1,00	1,00	0,99	0,99	1,00	1,00	1,00	1,00	1,00
glrlm_RunPercentage	0,99	1,00	0,99	1,00	1,00	1,00	1,00	1,00	1,00
glrlm_RunVariance	0,39	1,00	0,99	1,00	1,00	1,00	1,00	1,00	1,00
glrlm_ShortRunEmphasis	1,00	1,00	0,99	0,99	0,99	0,99	1,00	1,00	1,00

glrlm_ShortRunHighGrayLevelEmphasis	0,51	0,73	0,56	0,45	0,34	0,34	0,38	0,40	0,41
glrlm_ShortRunLowGrayLevelEmphasis	0,38	0,69	0,46	0,35	0,37	0,48	0,35	0,44	0,44
glszm_GrayLevelNonUniformity	0,88	0,98	0,90	0,90	0,88	0,85	0,88	0,89	0,89
glszm_GrayLevelNonUniformityNormalized	0,64	0,94	0,67	0,72	0,79	0,85	0,86	0,88	0,90
glszm_GrayLevelVariance	0,36	0,96	0,59	0,54	0,45	0,32	0,19	0,17	0,20
glszm_HighGrayLevelZoneEmphasis	0,49	0,71	0,56	0,45	0,34	0,33	0,36	0,38	0,38
glszm_LargeAreaEmphasis	0,98	1,00	0,99	0,99	1,00	1,00	0,99	0,99	0,99
glszm_LargeAreaHighGrayLevelEmphasis	0,05	0,09	0,35	0,20	0,05	0,04	0,06	0,09	0,09
glszm_LargeAreaLowGrayLevelEmphasis	0,68	0,36	0,37	0,24	0,21	0,44	0,27	0,30	0,22
glszm_LowGrayLevelZoneEmphasis	0,37	0,68	0,50	0,36	0,37	0,50	0,35	0,45	0,45
glszm_SizeZoneNonUniformity	0,93	0,99	0,94	0,94	0,95	0,95	0,95	0,95	0,95
glszm_SizeZoneNonUniformityNormalized	0,97	0,98	0,54	0,69	0,81	0,82	0,90	0,92	0,93
glszm_SmallAreaEmphasis	0,96	0,97	0,48	0,62	0,74	0,74	0,85	0,87	0,89
glszm_SmallAreaHighGrayLevelEmphasis	0,59	0,75	0,59	0,51	0,41	0,39	0,44	0,46	0,47
glszm_SmallAreaLowGrayLevelEmphasis	0,41	0,71	0,61	0,46	0,51	0,56	0,44	0,57	0,55
glszm_ZoneEntropy	0,44	0,93	0,80	0,77	0,75	0,73	0,83	0,89	0,91
glszm_ZonePercentage	0,97	1,00	0,98	0,98	0,98	0,98	0,99	0,99	0,99
glszm_ZoneVariance	0,98	1,00	0,99	0,99	1,00	1,00	0,99	0,99	0,99
ngtdm_Busyness	0,67	0,38	0,46	0,29	0,27	0,35	0,31	0,28	0,26
ngtdm_Coarseness	0,89	0,86	0,77	0,76	0,74	0,73	0,74	0,73	0,72
ngtdm_Complexity	0,26	0,87	0,59	0,57	0,51	0,44	0,43	0,42	0,47
ngtdm_Contrast	0,23	0,81	0,74	0,70	0,74	0,63	0,77	0,86	0,86
ngtdm_Strength	0,39	0,92	0,28	0,26	0,20	0,20	0,21	0,24	0,26
shape_Compactness1	0,48	0,78	0,70	0,70	0,70	0,70	0,70	0,70	0,70
shape_Compactness2	0,53	0,69	0,53	0,53	0,53	0,53	0,53	0,53	0,53

shape_Elongation	0,69	0,72	0,66	0,66	0,66	0,66	0,66	0,66	0,66
shape_Flatness	0,74	0,86	0,78	0,78	0,78	0,78	0,78	0,78	0,78
shape_LeastAxisLength	0,97	0,98	0,96	0,96	0,96	0,96	0,96	0,96	0,96
shape_MajorAxisLength	0,98	0,98	0,94	0,94	0,94	0,94	0,44	0,94	0,94
shape_Maximum2DDiameterColumn	0,71	0,90	0,86	0,86	0,86	0,86	0,86	0,86	0,86
shape_Maximum2DDiameterRow	0,96	0,96	0,95	0,95	0,95	0,95	0,95	0,95	0,95
shape_Maximum2DDiameterSlice	0,97	0,98	0,96	0,96	0,96	0,96	0,96	0,96	0,96
shape_Maximum3DDiameter	0,93	0,97	0,93	0,93	0,93	0,93	0,93	0,93	0,93
shape_MeshVolume	0,94	0,89	0,85	0,85	0,85	0,85	0,85	0,85	0,85
shape_MinorAxisLength	0,95	0,96	0,93	0,93	0,93	0,93	0,93	0,93	0,93
shape_SphericalDisproportion	0,25	0,89	0,81	0,81	0,81	0,81	0,81	0,81	0,81
shape_Sphericity	0,45	0,80	0,74	0,74	0,74	0,74	0,74	0,74	0,74
shape_SurfaceArea	0,43	0,97	0,85	0,85	0,85	0,85	0,85	0,85	0,85
shape_SurfaceVolumeRatio	0,27	0,75	0,77	0,77	0,77	0,77	0,77	0,77	0,77
shape_VoxelVolume	0,94	0,89	0,85	0,85	0,85	0,85	0,85	0,85	0,85
Median value	0,61	0,95	0,84	0,83	0,80	0,76	0,77	0,83	0,85
IQR 1	0,37	0,73	0,56	0,52	0,51	0,46	0,37	0,42	0,41
IQR 3	0,93	0,99	0,95	0,95	0,95	0,95	0,95	0,96	0,96
